# Emergence of scale-free characteristics in socio-ecological systems with bounded rationality

**DOI:** 10.1038/srep10448

**Published:** 2015-06-11

**Authors:** Dharshana Kasthurirathna, Mahendra Piraveenan

**Affiliations:** 1 Centre for Complex Systems Research, Faculty of Engineering and IT, The University of Sydney, NSW 2006, Australia

## Abstract

Socio–ecological systems are increasingly modelled by games played on complex networks. While the concept of Nash equilibrium assumes perfect rationality, in reality players display heterogeneous bounded rationality. Here we present a topological model of bounded rationality in socio-ecological systems, using the rationality parameter of the Quantal Response Equilibrium. We argue that system rationality could be measured by the average Kullback–-Leibler divergence between Nash and Quantal Response Equilibria, and that the convergence towards Nash equilibria on average corresponds to increased system rationality. Using this model, we show that when a randomly connected socio-ecological system is topologically optimised to converge towards Nash equilibria, scale-free and small world features emerge. Therefore, optimising system rationality is an evolutionary reason for the emergence of scale-free and small-world features in socio-ecological systems. Further, we show that in games where multiple equilibria are possible, the correlation between the scale-freeness of the system and the fraction of links with multiple equilibria goes through a rapid transition when the average system rationality increases. Our results explain the influence of the topological structure of socio–ecological systems in shaping their collective cognitive behaviour, and provide an explanation for the prevalence of scale-free and small-world characteristics in such systems.

Game theory is widely used to study and model strategic decision making scenarios, ranging from politics and market economics to ecosystems and information routing^1^,^2^,^3^,^4^. Network based games are increasingly used to understand critical phenomena in socio-ecological systems^5^,^6^,^7^,^8^,^9^,^10^,^11^. The concept of Nash equilibrium has been an important cornerstone in understanding the dynamics of such systems^12^. While Nash equilibrium assumes that all players in a system are fully rational, most real-world strategic decision making scenarios involve players with non-optimal or bounded rationality, resulting in their strategies and behaviour deviating from those predicted by the Nash equilibrium^13^. The possible limitations, such as the amount of information at hand, cognitive capacity, and the computational time available, may force a self-interested autonomous player or agent to have bounded rationality and therefore to make non-optimal decisions^14^.

Numerous theories have been presented to model the non-optimal rationality of players in strategic games, including the concepts of the *near-rationality equilibrium* and the *quantal response equilibrium*^15^,^16^,^17^,^18^. However, these models do not attempt to quantify, in a predictive manner, the levels of rationality prevalent in individual players based on their observable characteristics. Meanwhile, studies in psychology and cognitive science have conjectured that the rationality of individuals is correlated to the level of their social interactions^19^,^20^,^21^. In this paper, therefore, we propose a topological model of bounded rationality in socio-ecological systems, based on this conjecture. Using this model, we investigate how such systems could topologically evolve to have higher **system rationality**, given a heterogeneous bounded rationality distribution. Since the calculation of Nash equilibrium assumes perfect rationality of all players, we use the average Kullback–Leibler divergence between Nash and Quantal Response equilibria of each game played within the system as an indicator of overall **system rationality**. It is important to note that we distinguish this **system rationality** from the **average rationality**, which is simply the average of the heterogeneous rationality distribution of a system and therefore not influenced by its topology.

We show that among different topological classes of complex networks modelling socio-ecological systems, the scale-free class minimises this divergence (maximises the system rationality). Conversely, when a socio-ecological system with a random topology is optimised towards higher system rationality ( the system on average is driven towards Nash equilibrium), scale-free and small world features emerge. This result is true for games with single or multiple equilibria. In the case of games with multiple equilibria, the fraction of links in a network where multiple equilibria are actually prevalent is topologically dependent. We show that when average rationality is lower, the scale-freeness of the socio-ecological network aids in increasing the fraction of links with multiple equilibria. However, when the average rationality is higher, the scale-freeness actually aids in decreasing this fraction. In fact, we demonstrate that the correlation between the ‘scale-freeness’ and the fraction of links with multiple equilibria goes through a rapid transition when average network rationality is increased. Our results provide a possible explanation for the prevalence of scale-free features in the topologies of real world socio-ecological systems 22, and explore how the scale-freeness in turn affects the cognitive decision making behaviour of such systems.

## Background

### Complex Social Networks and networked game theory

In any cognitive decision making scenario played out in a socio-ecological system, the players are constrained by the contacts they create and maintain, and cannot arbitrarily interact with random players who have no direct ‘connection’ to them. Thus, these systems can be modelled as a complex network^22^. Most real world complex networks, including those representing socio-ecological systems, display two characteristic topological features: the ‘scale-free’ characteristic and the ‘small-world’ characteristic^22^,^23^,^24^,^25^,^26^. The scale-free networks display power-law degree distributions, while the small world networks are identified by relatively high clustering and relatively low average path length.

Game theory^2^,^27^,^28^,^29^ is an effective tool to model complex socio-ecological systems that involve multiple self-interested entities and decision making scenarios^30^,^31^,^32^,^33^,^34^,^35^. Over the past two decades, networked game theory has been increasingly used to understand the constrains placed by the social structure of a community on the cognitive decision making process of individuals^9^,^10^,^36^,^37^. In particular, the role of the scale-free structure has been well explored, typically in discussing its influence over the emergence of co-operation in games where the Nash-equilibrium occurs at mutual defection, such as Prisoners Dilemma^38^,^39^,^40^. A number of papers have suggested that the scale-free structure aids in the emergence of co-operation^8^,^38^. Other studies have, conversely, discussed the evolution of topological features in so-called co-evolutionary games where players are able to delete existing links and create fresh links to maximise pay-offs. It is often found that scale-free, or similar heterogeneous topologies (such as exponential) emerge as a result of such co-evolution. For example, Szolnoki *et al.*^40^ shows that in a Prisoners Dilemma game, networks of exponential degree distributions emerge when the network attempts to maintain cooperation as a viable strategy. In similar vein, there are other papers arguing that other node attributes, such as the ‘age’ of the participants^41^ or the ‘influence’ they have over other participants^42^, encourage cooperation best when these attributes are distributed heterogeneously (i.e following an exponential or power-law distribution). There are other studies, however, which argue that the non-uniform ‘participation costs’ involved in a heterogeneous social network negate the advantage the heterogeneity confers in encouraging cooperation - at least at an individual node level^43^. Yet other studies have argued that the scale-free nature of the social network has ‘sometimes enhanced and sometimes inhibited’ co-operation as a viable strategy^44^, listing out factors which decide the type of correlation. Meanwhile, a group of studies have found conditions, topological and otherwise, under which the number of people using a particular strategy (typically co-operation in the game of Prisoners Dilemma) go through a phase-transition (for e.g., see^45^,^46^).

In summary, the influence of heterogeneous network structure (particularly scale-free structure) on the survival of particular strategies has received considerable attention in recent times. It is in this backdrop that we set out to explore the relationship between network topology and the convergence towards Nash equilibria, which brings a fresh perspective in understanding the role of network structure in socio-ecological dynamics.

### Games between players with bounded rationality

The concept of Nash Equilibrium^12^,^47^ states that in a strategic decision making environment, there exists an equilibrium which no player would benefit deviating from. However, it has been observed that in experimental settings, the equilibrium states of players deviate substantially from those predicted by the Nash equilibrium^48^. One key reason for this deviation is the non-perfect, or bounded rationality of players.

Nash equilibrium assumes that the players always adopt the strategy that maximises their utility, and rationality is defined as the tendency to maximise one’s own utility under uncertainty^13^. However, in the real-world, the players may not be perfectly rational due to the limitations mentioned before^14^. Since these limitations vary from player to player, it is to be expected that the players would have heterogeneous bounded rationality, and would make some sub-optimal or apparently random decisions. A number of studies have indirectly modelled bounded rationality by introducing noise in strategies adopted by players (as done, for example, by^45^,^46^,^49^). However, the Quantal response equilibrium (QRE)^13^,^50^,^51^ directly presents an analogous way to model games with ‘noisy’ strategies, by using probabilistic choice model functions such as *logit* and *probit*^13^. These functions map the vector of expected payoffs from available choices into a vector of choice probabilities that is monotone with the expected payoffs. In statistical physics such functions are called Fermi functions^9^, and different versions of them have been used recently in studies involving spatial games^52^,^53^.

**Figure 1:**
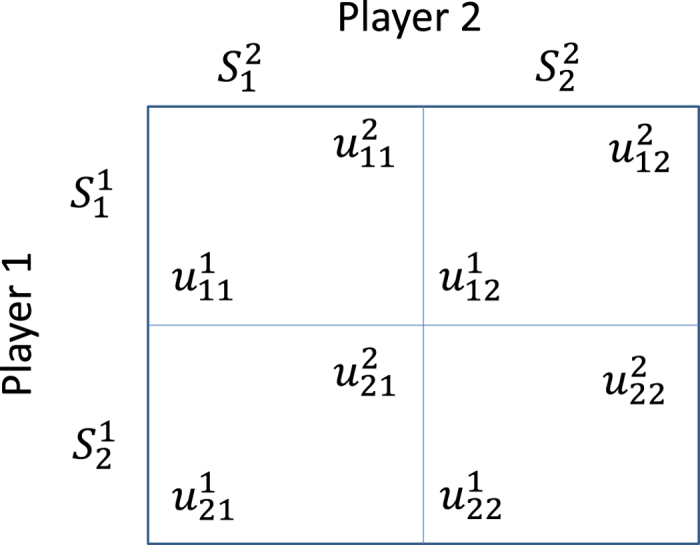
The payoff matrix of a generic normal-form game which involves two players. 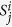 denotes strategy *j* adapted by player *i*, while 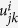 denotes the payoff to player *i*, when the first player adapts strategy *j* and the second player adapts strategy *k*.

Let us consider the payoff matrix of a generic normal form game (an example is given in Fig. 1 for two-player games). As shown in Methods, we choose to use the quantal response *logit* function (given in Eq.(3) in methods), to derive the Quantal Response Equilibrium of a player with a particular level of non-perfect rationality. The parameter *λ*_*i*_ in this equation is known as the **rationality parameter** of player *i*, and denotes the level of relative rationality the player *i* possesses, and can vary from zero to infinity. Thus, this parameter provides a convenient way to quantify the bounded rationality of a particular player and the resulting probability distribution denotes the quantal response equilibrium for that player at that particular bounded rationality level. The average of *λ*_*i*_ over all players, 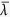, is thus an indicator of the average levels of rationality prevalent in the system.

### Relationship between rationality and social interaction

We argue that there is an implicit relationship between the amount of social interaction of a particular player and their bounded rationality. This argument, which is articulated by a number of studies, is critical in topologically quantifying the bounded rationality of players. For instance, the social cognitive theory^19^ suggests that knowledge acquisition is directly correlated to the observation of models. Thus, a player with a relatively higher amount of social interactions may have higher cognitive capacity compared to a player with a relatively lower amount of social interactions. The social learning theory^54^ expands on this concept. Similarly, the social brain hypothesis^20^,^55^ and studies extending it have established that there is a correlation between the human brain cortex size and the social cognitive capacity of humans^56^. This implies that increased social interactions aided in improving the cortex sizes, and thereby the cognitive capacity, of primates. Conversely, it could also be argued that the cognitive capacity of a person (player) is an ‘inherent’ property of a person, and the amount of interactions he engages in is rather a ‘reflection’ of that cognitive capacity. Either way, it is reasonable to argue that the cognitive capacity, i.e rationality, of a player is positively correlated to the amount of social interactions they undertake.

## Modelling

Although there have been attempts to model the rationality of players, they have mostly been concerned with proposing a rationality model that identifies the rationality as a constant for all players under a particular strategic decision making context. For example, Wolpert^17^ proposed a model to derive the rationality of an abstract player by solving the Maxent (maximum entropy) Lagrangians that model the probability distribution of a human player as a Boltzmann distribution. However, as the cognitive hierarchical model^21^ and related empirical observations suggest, rationality of players in a population is typically distributed in a heterogeneous distribution instead of all players having the same level of rationality for a particular strategic decision making environment. A social-interaction based modelling of bounded rationality would account for this heterogeneity.

Indeed, capturing the heterogeneity of rationality of players using the quantal response equilibrium has been studied before^18^,^57^, particularly with models such as Heterogeneous QRE model and Truncated QRE model^18^. It has been demonstrated that the cognitive hierarchical model^21^ is a special case of Truncated QRE model. However, these models too limit themselves to varying heterogeneous rationality parameter *λ*_*i*_ to fit the empirical results, modelling or applying it as an arbitrary parameter without any physical interpretation, while acknowledging that the rationality would be heterogeneous in a population of players. While this approach increases the versatility of the rationality parameter, it limits the predictive capacity of the QRE model. Therefore, we propose a model which has more predictive power, at least in relative terms for players within a population, as long as the assumption that the rationality of a player could be mapped ( by a linear or non-linear non-decreasing function) to their amount of social interaction is justified. The model we propose defines the rationality parameter *λ*_*i*_ for each player (node) as a function of social interactions.

At a very basic level, the number of social ties a player has, (i.e, the ‘degree’ of a node) could be an indicator of the amount of social interaction a player engages in. However, the amount of interaction would also depend on the ‘tie strength’ attributes, such as the amount of time spent, the volume of information exchanged etc, between each pair of players. Furthermore, the correlation between the amount of interaction of a player with other players, and the rationality of a player, could be linear or non-linear. To model such a dependency, therefore, we use a generic function *f*, to which the ‘weighted degree’ (on simply the ‘degree’, if tie strengths are considered equal) of a node is an input, as shown in Eq. (1).
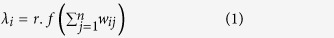
Here *λ*_*i*_ is the rationality of node *i*; *r* denotes a ‘network rationality parameter’ that would be a property of the network and represent the general level of rationality in the system. It should be noted that the average rationality of the system, 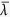, is proportional to this parameter, as any change in *r* will result in a corresponding proportional change in every *λ*_*i*_. The weight 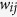 denotes the weight of the link connecting node *i* with each neighbour *j*, while *n* is the number of neighbours that node *i* has. In this work, we model the function *f* as simple linear, convex or concave functions, though in future studies empirical data could be used to fit a more accurate function for a given decision making context. The linear, convex and concave functions that we use are 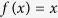, 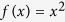 and 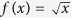 respectively, due to the simplicity and the computational efficiency of those functions and also due to the fact that they facilitate the 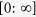 range of possible rationality values of the rationality parameter. Under this model, a node may behave completely randomly if the network rationality parameter is set to *r* = 0 or when the node is completely disconnected (ie. degree is zero). On the other hand, a node may make choices as predicted by the Nash equilibrium as the network rationality parameter 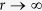, or when the degree of the node is extremely large.

### Analysis and Results

Using the topological model for bounded rationality presented by Eq. (1), we set out to answer the following questions: (i) Which topological features in a socio-ecological system facilitate the highest system rationality, given a particular heterogeneous rationality distribution among players? (ii) Is there a connection between the emergence of scale-free features in socio-ecological systems, and the need to optimise for better system rationality? (iii) Is there a connection between the emergence of multiple equilibria in systems with heterogeneous rationality, and the topological structure of such systems? In answering these questions, it is important to first define ‘system rationality’. Of course, the average of rationality parameters of all players in a system, 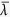, is one indicator for system rationality, however this definition disregards the topological effects. Therefore, we will use an independent measurement of ‘system rationality’ *ρ*, which is defined as the average Kullback–-Leibler divergence of Nash and QRE equilibria over all pairs of players, with a minus sign to account for the fact that the higher this divergence, the lower the system rationality. Details of the computation of *ρ* are given in the Methods. It is obvious that systems which have the same 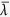 may have different *ρ*, since the later is topologically dependent.

### Comparing network topologies based on their average divergence from Nash equilibria

To answer the first question mentioned above, we chose three different network models for comparison: the scale-free network model, Erdös-Rènyi random network model and a regular (well-mixed) network model. The networks we analysed contained 500 or 1000 nodes with average degrees of 4, 6 or 8. We use the Prisoners Dilemma game in this analysis, since we choose to focus on games with a single equilibrium first. The payoffs used are given in Methods. The rationality of each node was calculated using the linear, convex and concave functions separately. Note that since we only need the equilibria, we did not actually simulate the games. Based on the Eq. (3), we derived the QRE for each pair of players (each link), as described in the Methods. The network rationality parameter *r* was set to 0.2, 0.002 and 0.5 for the linear, convex and concave functions, respectively. The single Nash equilibrium for Prisoners Dilemma occurs when both players defect. Therefore, once the QRE for each pair of players is obtained, the average KL divergence between Quantal Response and Nash equilibria for the network was computed, as described in the Methods.

Table 1 depicts a typical set of results. In this particular experiment, the number of nodes is *N *= 1000 and the number of links is *M *= 2000 (Note that in this and later experiments described, we have verified our results with much larger systems sizes, up to twenty thousand nodes and forty thousand links. The results show no qualitative difference and scale well. Some results for larger system sizes are included as supplementary material.). The simulations are done for two ‘game parameter’ values, *β*=1.33and *β*=1.67, as described in the methods section. The results are averaged over 100 instances, and in each instant, a *different* topology belonging to the same network class was used, while the number of nodes and links was kept constant. A degree-preserving re-wiring technique was used to create different ‘instances’ of the same topological class. All scale-free networks had a scale-free exponent of 2.0 with a 90% *R*^*2*^-correlation. According to the results given in Table 1, it is evident that the Nash-QRE divergence is minimum for the scale-free topology class under all three types of rationality functions. As one would expect, the convex rationality function gives the highest variations of average Nash-QRE divergence among different topology classes. The divergence is highest for the well-mixed topological class (We have verified that this result does not qualitatively depend on the utility values of the game, and increasing game parameter *β* in fact makes the difference between topologies more significant.). Thus, it is possible to conjecture that one reason for the prevalence of scale-free topology 22 in real-world socio-ecological systems in which strategic decision making takes place is that this topology facilitates the highest system rationality for a given heterogeneous rationality distribution among players.

### Optimising network topology for maximum system rationality

We observed that the scale-free topological class aids the convergence towards Nash equilibria on average, when compared to other topological classes. Conversely, we could observe whether evolutionary pressure on any given system that forces it to move towards Nash equilibria on average, while allowing the system to re-wire itself, results in the system becoming scale-free. Therefore, we undertook such topological optimisation using the Erdös-Rènyi random network class as the null model, and observed the resulting topological evolution. In order to perform the optimisation based on the convergence towards Nash equilibrium, we applied a variant of the *simulated annealing* technique^58^. Details are given in Methods. In this particular set of experiments, bounded rationality was measured using a convex function of degree (because the convex function 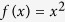 facilitated rapid topological evolution compared to the other functions) with the network rationality parameter being set to *r* = 0.1. We recorded the scale-free *R*^2^-correlation of each intermediate network, to observe the emergence of scale-free characteristics. Moreover, we recorded the clustering coefficient and the average path length of the intermediate networks, since these are the parameters that could be utilised to identify the small-world nature of the networks^22^. Relatively higher clustering coefficients and lower average path length are indications of small-world characteristics emerging^22^.

**Figure 2:**
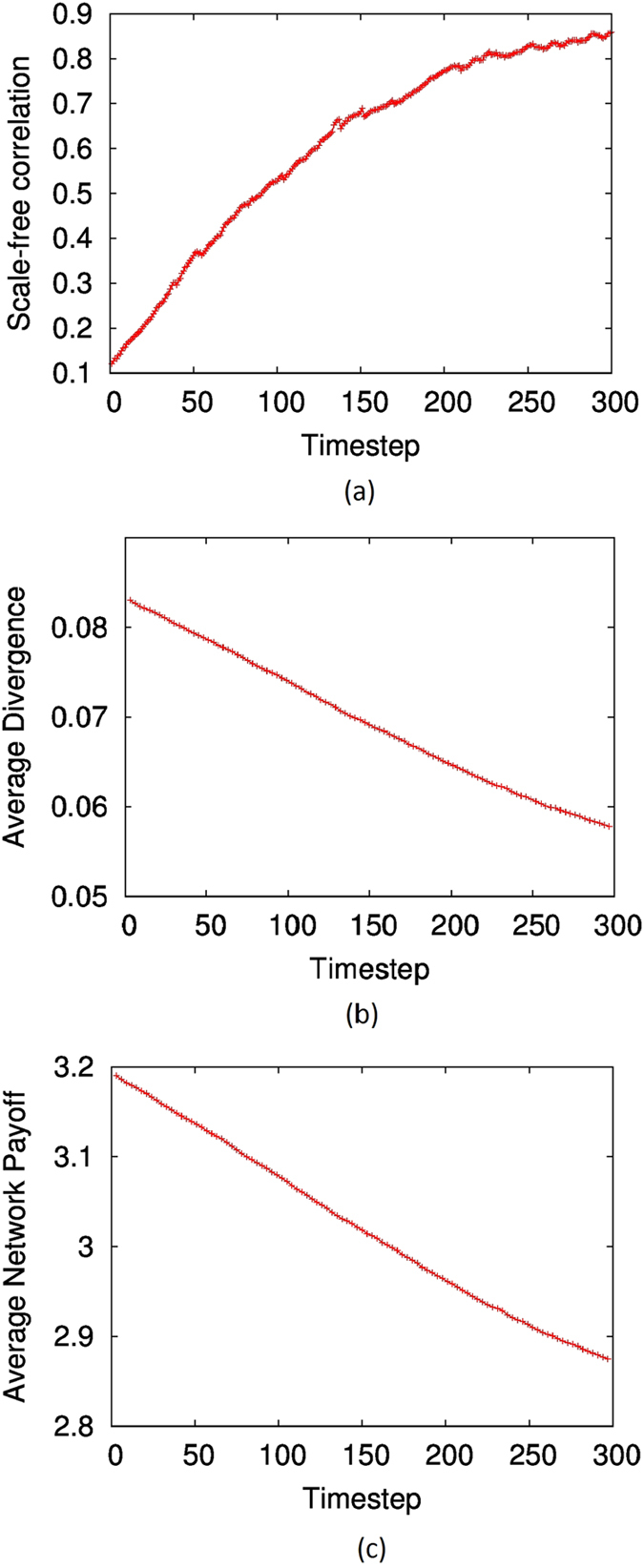
(**A**) The evolution of *R*^*2*^-scale-free correlation of a network over time, when it is optimised to minimise the average KL divergence between Nash-QRE equilibria, using simulated annealing. (**B**) The evolution of *−ρ*, average KL divergence between Nash-QRE equilibria, of the network over time. (**C**) The evolution of the average pay-off over time. Network size is *N* =1000, *M* =2000. The Prisoners Dilemma game was used in simulations.

**Figure 3:**
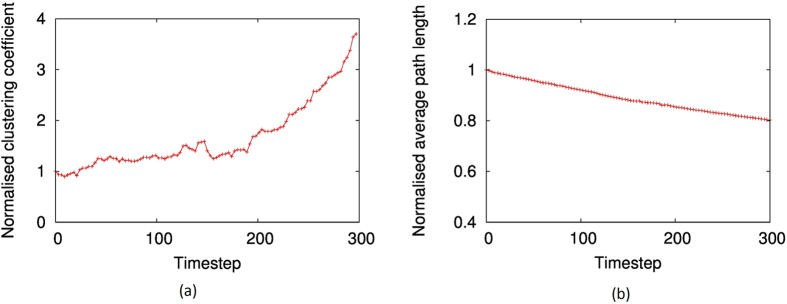
The evolution of the clustering coefficient and average path length of a network over time, when it is optimised to minimise the average KL divergence between Nash-QRE equilibria, using simulated annealing. The values are normalised by those of a random network of the same size. The Prisoners Dilemma game was used in simulations. Network size is *N* = 1000, *M* = 2000.

Typical results are shown in Fig. 2 and Fig. 3. As depicted by Fig. 2, the scale-free correlation shows an upward trend when the network evolves. Meanwhile as Fig. 3 shows, the clustering coefficient clearly increases while the average path-length decreases over time, indicating that the ‘small-worldness’ also increases over time. Even though these results are for networks with size *N* = 1000 and *M* = 2000, we obtained similar results for networks with average degrees 4,6 and 8, and *N* = 500, attempting all possible permutations. From these results, it is clear that when network topology is optimised towards maximum system rationality (i.e converegence towards Nash equilibria on average is favoured), scale-free and small-world features emerge in systems with random topology.

Interestingly, we also find that the average trend towards system rationality does not imply that the average pay-off for the players will also increase. In fact, for Prisoners Dilemma at least, the converse is true, as Fig. 2 indicates. However, we need to bear in mind that the premise behind Nash equilibrium in Prisoners Dilemma is that given the uncertainty about the other player’s decision, each player will make a selfish decision which would ensure that they are not worse-off than the other player, even though the expected utility of that decision is lower than both co-operating. That is indeed the ‘dilemma’. The drive towards Nash equilibria in this case does not imply an increase in the ‘public good’ of the system^59^. We will discuss this point further in section.

### Emergence of scale-freeness in games with multiple equilibria

So far, we primarily focused on games with single pure Nash equilibria. However, most normal-form games consist of multiple pure and mixed Nash equilibria. When we assume that the bounded rationality of a population of players is heterogeneous, the existence of multiple equilibria adds an extra layer of complexity. In order to observe how the rationality parameter would affect the quantal response equilibria in a game where there exists multiple equilibria, we used a set of coordination games that have two pure Nash equilibria. These included (i) the stag-hunt game, where the two pure Nash equilibria occur when either both players coordinate or both players defect (ii) the meeting game, where the Nash equilibria occurs when both choose one location or when both players choose the other location to meet (iii) The matching-pennies game^60^, where the Nash equilibria occurs when the symbols on the penny each player comes up with (head/tail) do not match.

We begin with the stag-hunt game, with typical payoffs as described in Methods, and follow an optimisation process using simulated annealing, again as described in Methods. Note that since there are multiple equilibria, the *lowest* divergence for each pair of players was used to compute the average KL divergence. Again, we recorded the scale-free *R*^2^-correlation of each intermediate network, to observe the emergence of scale-free characteristics.

**Figure 4:**
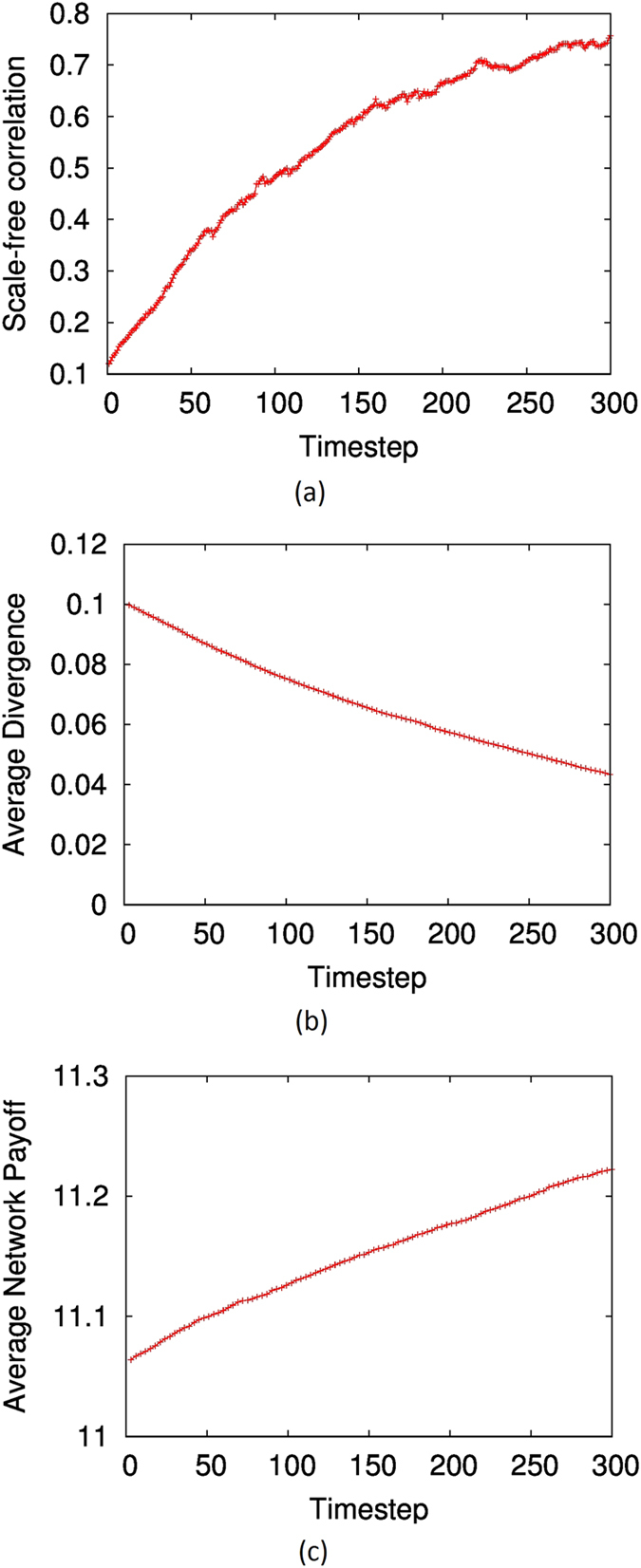
(**A**) The evolution of the *R*^*2*^-correlation to power-law degree distribution (scale-freeness) of the network over time, when it is optimised using Nash-QRE KL divergence and simulated annealing. (**B**) The evolution of the average Nash-QRE KL divergence of the network (*−ρ*) over time. (**C**) The evolution of the average pay-off over time. Network size is *N* = 1000, *M* = 2000. The stag-hunt game was used in simulations.

As depicted by Fig. 4[A], the scale-free correlation shows an upward trend when the network evolves. The corresponding average divergence −*ρ* is shown in Fig. 4[B], which, as expected, is minimised during the process. Interestingly, the average-payoff of players, shown in Fig. 4[C], increases as well. This is in contrast to the Prisoners Dilemma game, where the increase in ‘selfish-rationality’ of the system, represented by *ρ*, does not increase the average pay-off. As noted before, depending on the nature of the game, the average ‘selfish rationality’ corresponds to the ‘public good’ in some games, and does not in others. Regardless, the main observation that the topological optimisation towards high system rationality results in the emergence of scale-free characteristics is true also for games with multiple equilibria. We further confirmed this by conducting experiments with other two-player games, such as the meeting game (also called the battle of the sexes) and matching-pennies. We avoid showing the results here, which are qualitatively similar.

We also recorded the average clustering coefficient and the average path length during this optimisation process, for all the above mentioned games. We observed that, similar to the results obtained for Prisoners Dilemma, the average path length decreases and, the average clustering coefficient increases, when the network is optimised towards higher system rationality. To avoid repetition of similar results we avoid showing these results here. In summary, all these results confirm that when a network is topologically optimised towards increased system rationality, scale-free and small-world features emerge for a range of single and multiple equilibria games. Conversely, we also verified (not shown) that among the three topological classes we considered (scale-free, random and regular), it is the scale-free class which showed the highest system rationality *ρ* for all the above-mentioned multiple-equilibria games.

### Network topology and fraction of links with multiple equilibria

**Figure 5:**
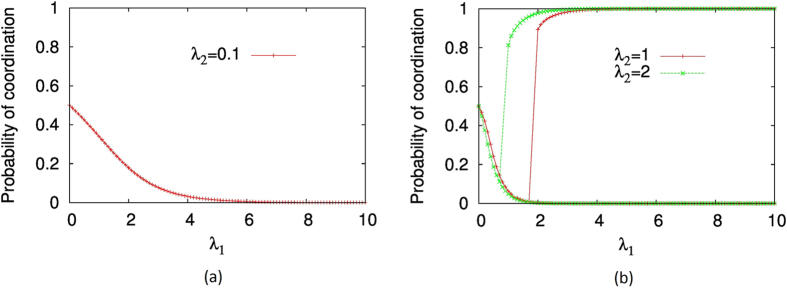
The variation of player 1’s coordination probability when the rationality parameter of player 2 is fixed (at either 0.1,1.0 or 2.0) and the rationality parameter of player 1 is varied. When the rationality of the opponent is small, there is only one equilibrium and otherwise, there are multiple equilibria. The stag-hunt game was used. Note that the *λ* values picked (0.1,1.0,2.0) have no particular significance other than illustrating the contrast of multiple equilibria occurring/not occurring.

Now we turn our attention to the actual prevalence of multiple equilibria in games in which multiple equilibria are possible, and how the interplay between heterogeneous rationality and network topology influences this prevalence. In particular, we compute the fraction of links with multiple equilibria in the landscape defined by varying scale-freeness and varying average rationality, indicated by the network rationality parameter *r*. It has been previously claimed that in two-player games, the players go through transitions of knowledge of opponents when the rationality parameter increases^61^. To begin with, we consider a single pair of players playing stag-hunt and verify that the rationality of both players would influence the number of multiple equilibria in the system. We solve the quantal response equilibria equations, for a range of *λ*_*i*_ values for both players, as described in Methods. Figure 5 depicts the results observed. For a given player, when the opponent’s rationality is relatively high (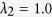 or 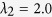), multiple equilibria can exist, and the probability of coordination goes through a transition, as predicted by Harre *et. al*.^61^. If the rationality of the opponent is relatively low (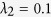), such transition does not occur. Therefore, we can verify that for a single pair of players, multiple equilibria does not always occur and that the rationality levels of both players influence whether there could be multiple equilibria. Hence, it is clear that in a socio-ecological system (represented by a complex network) with a heterogeneous rationality distribution, on which a multiple-equilibria game is played, only a fraction of links would actually support multiple equilibria.

Now let us consider a socio-ecological system of players with a heterogeneous rationality distribution who engage in such a game with multiple equilibria, and analyse how the system topology (particularly the level of scale-freeness) would influence the number of multiple equilibria. Therefore we generated a range of scale-free networks with varying scale-freeness and identical size *N* = 1000 and *M* = 2000. To do this, we first generated perfect scale-free networks using the well-known Barabási-Albert model^22^, and introduced a measure of ‘randomness’ in each network by randomly rewiring *m* <*M* number of links. We varied *m*, and measured the ‘scale-freeness’ of each resulting network by fitting a power-law degree distribution and measuring the fitness, as mentioned before. For each of these networks, we used Eq. (1) to generate a heterogeneous rationality distribution, and then for each pair of nodes, computed the QRE equilibria as shown in Methods. Then we counted the links which would have multiple (in this case, two) equilibria, and finally thus computed the proportion of links in the entire network which had multiple equilibria. For this experiment, we used a convex rationality function. We repeated the whole process for different network rationality parameter values, beginning from *r* = 0.01 (low average rationality) to *r* = 0.3 (high average rationality).

**Figure 6:**
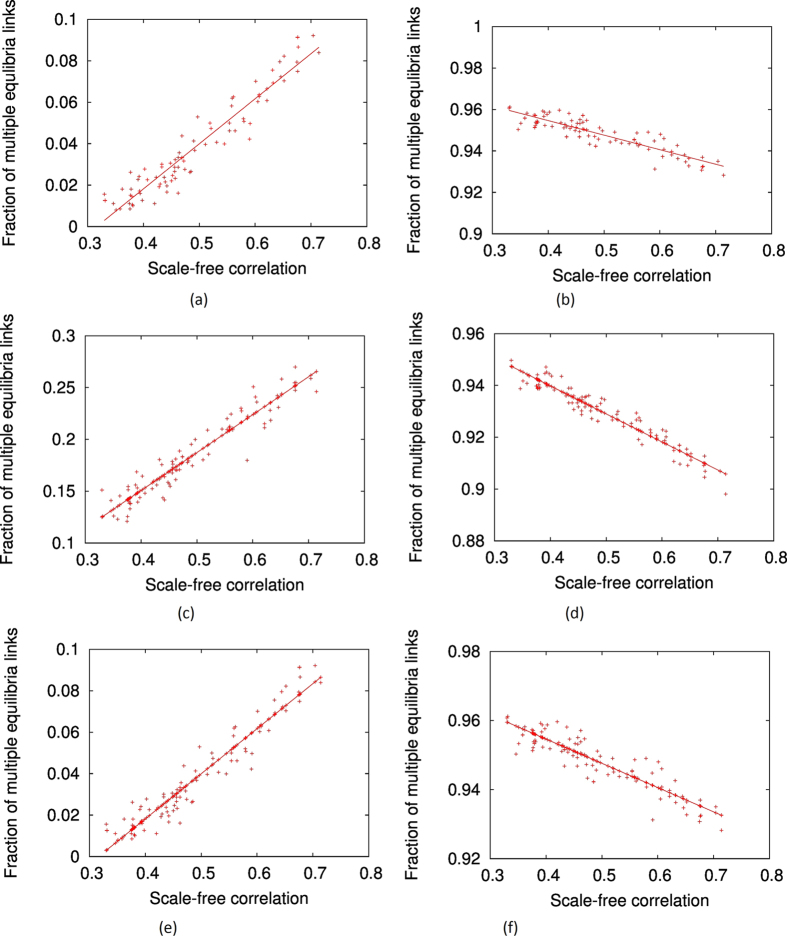
The variation of the fraction of links in games with multiple equilibria of networks with varying scale-freeness, under smaller (0.01) and larger (0.30 or 0.10) network rationality parameter values. (**a**) Stag hunt, r = 0.01 (**b**) Stag hunt, r = 0.30 (**c**) Battle of the sexes, r = 0.01 (**d**) Battle of the sexes, r-0.10 (**e**) Matching pennies, r-0.01 (**f**) Matching pennies, r = 0.30. The results for the stag-hunt game, battle of the sexes and matching pennies games are shown.

**Figure 7:**
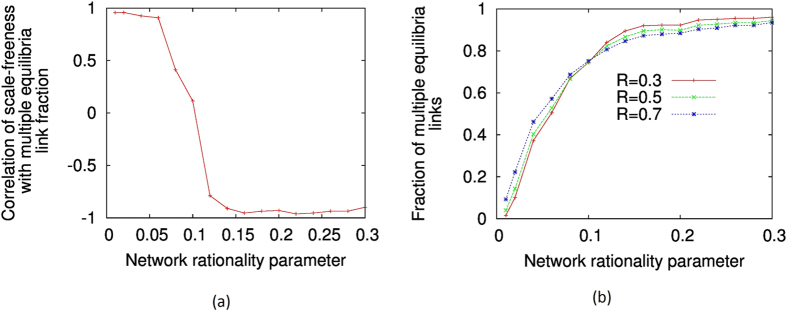
The variation of the fraction of links with multiple equilibria of networks with varying scale-freeness, under smaller (0.01) and larger (0.30 or 0.10) network rationality parameter values. The results for the stag-hunt game, battle of the sexes and matching pennies games are shown.

**Figure 8:**
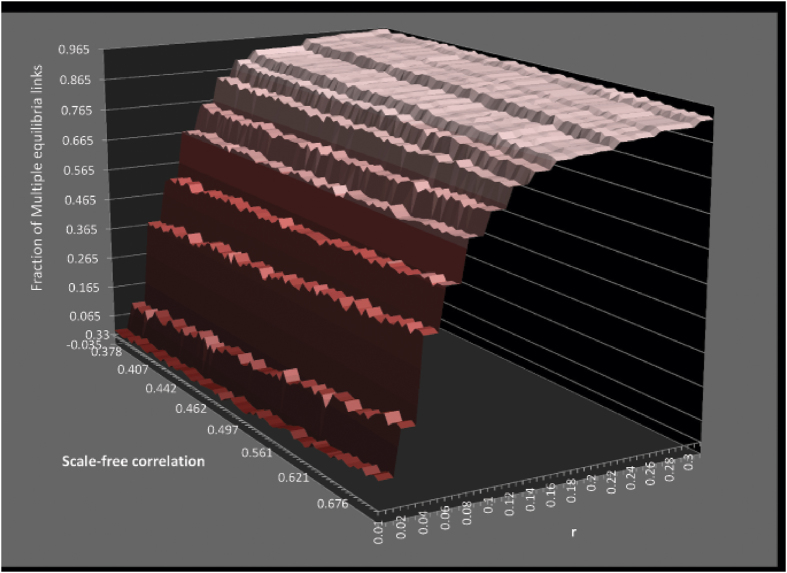
A three-dimentional plot showing the fraction of links with multiple equilibria against ‘scale-freeness’ (*R*^*2*^-correlation) and network rationality parameter *r*, for the stag-hunt game. Please note that the apparent stratification is simply a result of the limited number of *r* values used. To increase clarity of the figure, we only show a section of the scale-freeness range we have used.

We show our results for two particular values of network rationality, *r* = 0.01 and *r* = 0.3, in Fig. 6[a] and Fig. 6[b], for the stag-hunt game. In these figures, we plot the fraction of links where multiple equilibria is possible against the ‘scale-freeness’ of the network, represented by the scale-free *R*^2^-correlation. Eighty different networks of increasing scale-freeness have been shown in each plot. We may see that for relatively lower network rationality (*r* = 0.01), the ‘scale-freeness’ of networks have clear positive correlation with the fraction of links with multiple quantal response equilibria. That is, when the scale-free nature of the network increases, it becomes easier for links to attain multiple equilibria. By contrast, when the network rationality is relatively high (*r* = 0.3), the fraction of links with multiple equilibria has a negative correlation with the ‘scale-freeness’ of the network. That is, the emergence of scale-freeness encourages single equilibria among the pairs of players in the population. In fact, to generalise this result, we could compute the correlation between the fraction of links with multiple equilibria and the ‘scale-freeness’ (*R*^2^-correlation) of the network for several network rationality parameter (*r*) values, and expect to see a value of *r* between *r* = 0.01 and r = 0.3 where this correlation changes sign. Figure.7[A] depicts the results of such an experiment, where 16 different values of *r* from *r* = 0.01 to *r* = 0.3 are used. From this figure, we can see that indeed such a change of sign occurs when 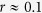. Moreover, this change of sign is not gradual but appears to undergo a rapid transition. Note that the same set of 80 scale-free networks were used to generate each data point in this plot, with differing values of *r*. Thus, the correlation of ‘scale-freeness’ with the fraction of links with multiple quantal equilibria goes through a transition when the overall network rationality, represented by the network rationality parameter *r*, is increased. Let us note, however, that by ‘transition’ we only denote a non-linear rapid increase and do not necessarily claim this to be a phase transition. The concept of phase transition has a very specific meaning in statistical mechanics which is used rigorously in evolutionary game theory^62^, and a handful of studies have observed such transitions within the context of spatial evolutionary games^63^,^64^. On the other hand, as Fig. 7[B] shows, for the same level of ‘scale-freeness’ the fraction of links with multiple equilibria increases with rationality parameter *r*. This is also confirmed by Fig. 6 which shows that the *range* of fraction values is much higher when *r* = 0.3 compared to *r* = 0.01. We summarise all these results in a 3D plot (Fig. 8), which shows the fraction of links with multiple equilibria in a stag hunting game for a range of scale-free networks with differing ‘scale-freeness’ and rationality parameter *r*. The dominant trend shows the fraction increasing then stabilising against rationality; however, we can also note the positive correlation with scale-freeness for lower *r* values and the negative correlation with scale-freeness for higher *r* values. These correlations are relatively less visible however, and it is for this reason we showed them separately in Fig. 6 which clearly indentifies the correlation tendencies.

These results are vital in understanding the relationship between network topology and cognitive decision making in systems with bounded rationality. They suggest that when the socio-ecological system as a whole has less average rationality (i.e more likely to make random decisions), the scale-free structure of the system helps players to have a higher number of rational choices. Yet, if the system becomes more rational on average, the same scale-freeness becomes a hindrance to players having a higher number of rational choices. If a society is increasingly becoming ‘selfishly wise’ (i.e, rational in the game theoretic sense), there will come a time where a slight change in the average rationality will have a huge bearing in the number of rational choices the players may have. In this set of experiments, when *r ≈ 0.1*, slight changes in rationality will hugely impact the fraction of multiple equilibria. Finally, the observations that we made suggest that even though a particular strategic decision making scenario may potentially encompass multiple equilibria, the actual prevalence of multiple equilibria in a population is topologically dependent and connected to the average rationality of that population.

To verify whether the results observed were specific to the stag-hunt game or could be generalised to other games with multiple equilibria, we undertook similar experiments with the meeting game (battle of the sexes) and the ‘matching-pennies’ game described earlier. we used the same set of scale-free and partially scale-free networks which we used in the experiments with stag-hunt game. The typical results are shown in Fig. 6[Parts C,D,E, and F]. These figures indicate that the observations made earlier are generic and not specific to the stag-hunt game alone, and that in any game with multiple equilibria, the scale-free features facilitate the prevalence of multiple equilibria when average network rationality is lower, and hinder the prevalence of multiple equilibria when average network rationality is higher.

## Discussion

The presented analysis resulted in significant findings. First of all, we compared a number of network classes, including scale-free, Erdös-Rènyi random, and lattice networks (representing well mixed populations), and showed that among these classes, it is the scale-free networks which facilitate the best convergence towards Nash equilibrium (highest system rationality), on average. We argued that this might be one reason why many real-world social systems are scale-free. Seeking further evidence for this conjecture, we simulated the topological evolution of social systems using the simulated annealing technique, beginning from a random network topology. We showed that when evolutionary pressure is applied on social systems to converge, on average, towards Nash equilibria, scale-free and small world features emerge. This is a very significant finding, since it provides an alternative explanation for the prevalence of scale-free networks in many real world systems and societies.

Following this, we turned our focus on games with multiple equilibria. Again, we demonstrated that when evolutionary pressure is applied on systems to converge, on average, towards Nash-equilibria (regardless of which equilibrium state a particular pair of players converge towards), scale-free and small world features emerge. We also considered the likelihood of the existence of multiple equilibria among the players of a system with a bounded heterogeneous rationality distribution, and found that a delicate balance exists: when the average rationality (this must be distinguished from what we call the ‘system rationality’, which is computed from the KL divergence between QRE and Nash equilibria) is low, the scale-free nature of the system encourages the emergence of multiple equilibria, while when the average rationality is high, the scale-free character in fact hinders the existence of multiple equilibria. Therefore, the number of rational choices available to players, from which they cannot deviate without loss, depends on the social network topology as well as the level of rationality prevalent in the system.

It is important to understand that ‘rationality’ of players and that of a system have been defined in a very specific way in our work. It could be argued that ‘rational’ players are those who try to maximise their average individual pay-offs. If players attempted to do this within a heterogeneous system, they may well make choices that are contrary to Nash equilibrium. Therefore, a system which converges towards Nash equilibrium will not necessarily have increasing average pay-offs. Indeed, in the case of Prisoners Dilemma game, the convergence towards Nash equilibrium results in decreasing average pay-offs. Thus, it could be argued that such a system is, on average, not becoming more ‘rational’. However, in an environment where there is a lot of ‘mistrust’ and/or competition, the priority of the players will be to make sure that their average pay-offs are better than other players with whom they compete - that is, they would want to ensure that they are not ‘cheated’ by others. The self-interest, and the relative well-being in the system, therefore gains prominence over the ‘absolute well being’, represented by the cumulative pay-off. In such systems, the convergence towards Nash equilibria, on average, means the players are getting better at preserving their ‘relative’ self-interest, and thus becoming more ‘raional’ in a selfish sense. The findings we present here are related to this sense of rationality, and not the ‘public good’ of the system^59^. However, in games other than Prisoners Dilemma (for example, in the stag-hunt game), we find that the average pay-off indeed could increase as the system converges towards the (multiple) Nash equilibria, depending on the actual values of pay-offs for each scenario. Thus, the ‘public good’ of the system matches with the selfish rationality of players. Therefore, it is important to realise that the results we have obtained are applicable in terms of average selfish rationality of players, which sometimes matches with the ‘public good’ of the system and sometimes does not.

In summary, it is a vital research endeavour of great scientific and practical significance to understand how the cognitive decision making of players and the resultant dynamics in socio-ecological systems are shaped by both the topology of such systems and the bounded rationality of actors in such systems.

## Methods

### Description of two-player normal form games

A number of classical games are typically used to study cognitive behaviour of players in complex socio-ecological systems^5^,^5^,^65^. Since these games in their simplest forms are well known and understood, let us just mention the exact pay-off values we used in our experiments. We only use normal form games. The meanings of the symbols are as per Fig. 1.

*The prisoner’s dilemma (PD)*^38^,^39^,^65^: 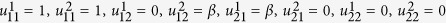. The game parameter *β* is fixed at 1.67 unless otherwise stated. There is a single pure Nash equilibrium.

*The stag-hunt* 3,39,49: 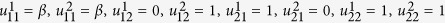. The game parameter *β* is fixed at 1.67 unless otherwise stated. There are two pure strategy Nash equilibria.

*The meeting game (The battle of the sexes)* 3,66: 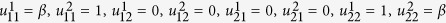. The game parameter *β* is fixed at 1.67 unless otherwise stated. Again, there are two pure strategy Nash equilibria.

*The matching-pennies game:* As an example of an asymmetric game, we use a version of what is generically described as a ‘matching-pennies’ game^60^. Here 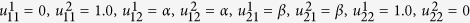. The game parameters *α,β* are fixed at 2.5, 2.0 respectively unless otherwise stated, and always 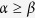. There are two pure strategy Nash equilibria (though in the classical *symmetric* matching pennies game, there are no pure strategy equilibria).

Note that in all cases the game parameters must be greater than one (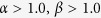) for the pay-offs to be in intended order.

### Network Models

*Scale-free Networks:* Scale-free networks retain similar topological characteristics irrespective of the scale. Many social networks are scale-free and heterogeneous, because there are always people who are more ‘famous’ and well-connected, while there are many who are relatively isolated. Scale-free networks display power-law degree distributions, described by 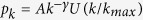 where *U* is a step function specifying a cut off at 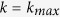. There are a number of growth models which generate scale-free networks, and prominent among them is the Barabási-Albert model^22^ utilising preferential attachment, which we use in this work. Due to the prevalence of scale-free features in many online and offline social networks, scale-free networks are good models to study games on social systems, and often used for this purpose in recent literature^39^.

To quantitatively measure the ‘scale-freeness’ of a particular network, we use the *R*^*2*^-correlation of the degree distribution to a power law. To compute this, we plot the degree distribution of the given network (in log-log scale) and fit a straight-line to this distribution (in the form of 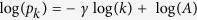 ) and then compute the *R*^*2*^-correlation (also called the *correlation of determination*) of the fit. The *R*^*2*^-correlation is computed as:
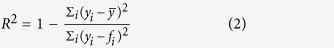
where *y*_*i*_ are y-values of the data points, 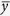 is the mean y-value of the data points, and *f*_*i*_ are the values returned by the fitted function for data points *i*^67^. This quantity is briefly called ‘scale-free correlation’ elsewhere in the text, to mean that it is the *R*^*2*^-correlation measuring the scale-freeness of a given network.

*Small-world Networks:* Small-world network model suggests that despite the large network size, the average distance between two arbitrary nodes remains relatively low^22^. Small-world networks have low characteristic path lengths (compared to network diameter) and high clustering(68,69,70). It has been shown that a range of real-world networks, including social networks, biological networks such as Gene Regulatory Networks, metabolic networks, Protein-Protein Interaction networks, and signalling networks, as well as the Internet show the small-world property^22^,^71^. Of course, many small-world networks can be scale-free to a certain degree, and vice-versa, but the scale-free and small-world characteristics need not (and often, do not) overlap^49^.

*Other network models:* We also make use the Erdös-Rènyi random topology 22 as a null model, and sparse lattice networks as an approximation for well-mixed populations^39^,^49^,^72^.

### Computing the Quantal Response equilibrium for a pair of players

We use the *logit* function given in Eq. (3) for computing the Quantal Response equilibrium, as often done in literature^13^,^73^.
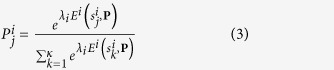
Here, 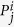 is the probability of player *i* selecting the strategy *j*. 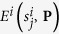 is the expected utility to player *i* in choosing strategy *j*, given that other players play according to the probability distribution **P** (which is also denoted 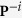 in some literature to highlight the fact that entries ‘belonging’ to player *i* should be discounted when the other players are considered collectively). The total number of strategies that player *i* can choose from is given by *K*. The rationality parameter *λ*_*i*_ can vary from zero to infinity. It can be shown that as 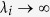, the equilibrium probabilities tend towards those given by the Nash equilibrium, and as 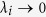, the player would operate in a totally random (irrational) fashion^13^.

For a two-player Prisoners Dilemma game, we can derive Eq. (4) and Eq. (5) from Eq. (3) to represent the probabilities that the two players would co-operate.
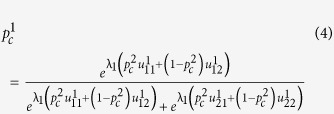

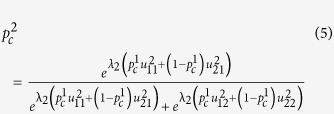
Here, 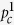 and 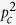 are the probabilities player 1 and 2 would co-operate, respectively, and *λ*_*1*_, *λ*_*2*_ denote the rationality parameters of player 1 and 2, which are derived using their respective degrees, the rationality function and the network rationality parameter used, as prescribed in Eq. (1), with identical link weights set to unity. The two equations have two unknowns 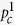 and 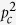. Thus, we can solve these two equations and calculate the probability of cooperation ( and defection ) for a particular pair of players and rationality parameters. The resulting probability distributions provide the QRE for the particular pair of players.

We already know that the only Nash equilibrium for this game would occur when both players defect (that is, 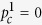, 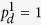 and 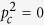, 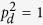).

We followed a similar procedure to calculate the QRE for other games used in this study, using the respective set of utilities for each game as mentioned earlier in Methods.

### Computing system rationality as an average Kullback–Leibler divergence between Nash and Quantal Response equilibria

The Kullback–Leibler (KL) divergence^74^, also known as the relative entropy, is commonly used to measure the distance or the divergence between two probability distributions. We use the ‘average’ KL divergence between the Quantal Response and Nash equilibria as an indicator of the system rationality, based on the assumption that the more ‘selfishly rational’ the players are on average, the closer they will be to Nash, and the less this divergence will be. Given two probability distributions *P* and *Q*, the KL divergence between them is calculated by,
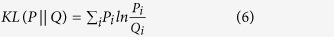
where *P*_*i*_ and *Q*_*i*_ are elements of the distributions in concern, respectively. However, the Kullback–Leibler (KL) divergence is an asymmetric measure, whereby the distance from P to Q is not the same as that from Q to P. Where a symmetric measure is more appropriate, an ‘average’ of the divergence is often used in the literature, which is sometimes known as Jensen-Shannon divergence^75^. This ‘average’ is computed as
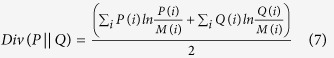
where *M* is the distribution computed by averaging the two probability distributions P and Q. It is this metric that we use in this work. This is then further averaged across all links, as shown in Eq. (8). Note well therefore that the ‘averaging’ is done at two levels. The ‘system’ rationality *ρ* is given by Eq. (8), where the negative sign indicates that the lower this divergence is, the higher the average system rationality. Note that in Eq. (8), *N*_*K*_ represents the probability distribution of the Nash equilibrium at link *k*, and *Q*_*k*_ similarly represents the probability distribution of the quantal response equilibrium at link *k*, and there are *M* links in the socio-ecological network in total.
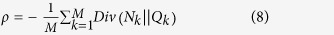
This average KL divergence should not be confused with the average rationality parameter, 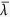, of the system. The 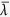 indicates the average of the heterogeneous rationality distribution, but two systems with same 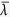 may show different average KL divergence from Nash equilibria, because of the topological placement of each player in those systems.

### Simulated annealing optimization

The process began with an Erdös-Rènyi random network. In each iteration, 0.33% (i.e M/300) randomly selected links were rewired so that the average KL divergence from Nash equilibrium decreases. The iterations were continued until *M* rewirings were made in total (i.e three hundred iterations). For each intermediate network, the scale-free *R*^*2*^-correlation, the average clustering coefficient and the average path length were recorded. The definition and computation of these metrics is well-understood and we avoid repeating them here^22^. If there are multiple equilibria, the *lowest* divergence for each pair of players was used.

## Additional Information

**How to cite this article**: Kasthurirathna, D. and Piraveenan, M. Emergence of scale-free characteristics in socio-ecological systems with bounded rationality. *Sci. Rep.* **5**, 10448; doi: 10.1038/srep10448 (2015).

## Supplementary Material

Supporting Information

## Figures and Tables

**Table 1:** The average Nash-QRE divergence (*−ρ*) of network topologies for different rationality functions.

***β*****=1.33**	**Scale-free**	**Random**	**Well-mixed**	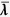
**Linear**	0.378	0.396	0.408	0.8
**Convex**	0.425	0.430	0.431	0.008
**Concave**	0.383	0.392	0.403	2.0
***β*****=1.67**	**Scale-free**	**Random**	**Well-mixed**	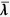
**Linear**	0.338	0.367	0.383	0.8
**Convex**	0.419	0.427	0.429	0.008
**Concave**	0.345	0.360	0.363	2.0

The rows represent the rationality functions while the columns contain the topology of the population. The network rationality parameter *r* was set to 0.2,0.002 and 0.5 for the linear, convex and concave functions, respectively. The average rationality parameter of nodes, 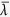 is also shown. Note that the results were further averaged over 100 different instances of the same topological class. Two ‘game parameter’ values *β* = 1.33 and *β* = 1.67 are used, as detailed in methods.
