# Supplementary material for: Emergence of scale-free characteristics in socio-ecological systems with bounded rationality
Source: Sci Rep. 2015 Jun 11;5:10448. doi: 10.1038/srep10448 (PMC4464151; doi:10.1038/srep10448)
Supplement: Supporting Information [file srep10448-s1.pdf]

Emergence of scale-free characteristics in socio-ecological systems with bounded rationality

Supplementary material

Dharshana Kasthurirathne and Mahendra Piraveenan

This document provides some supplementary information, including additional results which could not be included in the paper due to space restrictions, and further explanation regarding certain computations and methods.

Emergence of scale-free features: degree distributions

In Fig.2 in the paper, we show evidence that scale-free networks emerge when topological changes are made to minimise the average Kullback-Leibler divergence between Nash and quantal response equilibria in the system. Here we show the corresponding degree-distributions at the start and end of the simulation, in Fig. 1. It can be clearly seen that at the beginning the system shows a random topology with near-Poisson degree distribution, whereas at the end of the simulation the system shows scale-free characters with degree distributions resembling a power-law. The correlation of determination (The R^2 correlation) when a power-law is fitted to this distribution is measured and shown in Fig.2 of the paper. Note however that Fig.1 corresponds to a single run of a simulation, and not the average of twenty individual runs as shown in the paper.

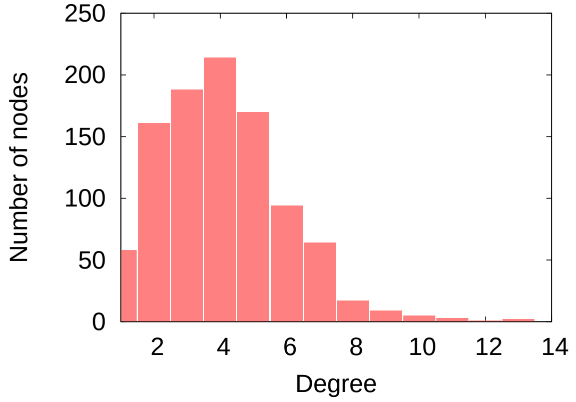

(a)

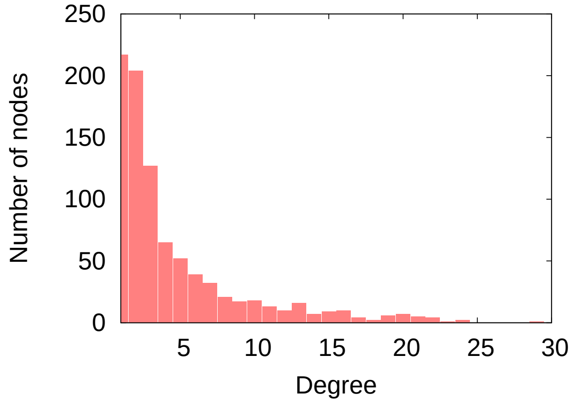

(b)

Figure 1: (a) The degree distribution of the initial network (b) The degree distribution of an evolved network under simulated annealing after 300 timesteps. Network size is $N = 1000, M = 2000$. The Prisoners Dilemma game was used in simulations. Note that the evolved network shows power-law characteristics.

Emergence of scale-free features in large systems

Further experiments were carried out to confirm that the result reported in the paper (that scale-free networks emerge when topological changes are made to minimise the average Kullback-Leibler divergence between Nash and quantal response equilibria in the systems) scales well and is true for larger systems. Whereas the system sizes shown in the paper are $N = 1000$, $M = 2000$, we have simulated systems of sizes up to $N = 20000$ nodes and $M = 40000$ links. Some results are shown in Fig. 2. Again, those shown in Fig. 2 are results from a typical run of experiment and not averaged over twenty runs as shown in the paper, yet the trend is smooth enough. The figure confirms that the increasing trend in the 'scale-freeness' (as measured by the R^2 correlation of power-law fitted to the degree distribution) is true for larger system sizes as well. We obtained similar results which confirmed increasing 'small-worldness' in larger system sizes in a similar context.

Comparison of network topologies using parameterised game models

In the paper, we typically model our games (such as Prisoners Dilemma or Stag-hunt), using a 'game-parameter' β, which is typically the ratio between the highest utility and the second highest utility in a given game. For example, in stag-hunt if both players hunt stag they each receive β, while if one hunts rabbit he receives 1.0. This modelling was used rather than the traditional pay-off values to demonstrate that the results we report do not depend on fixed-value game models.

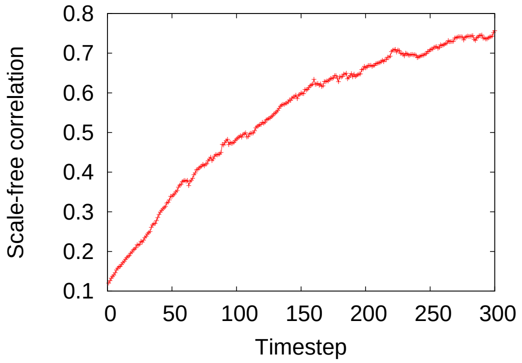

(a)

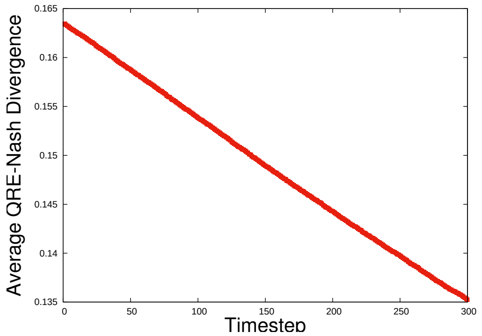

(b)

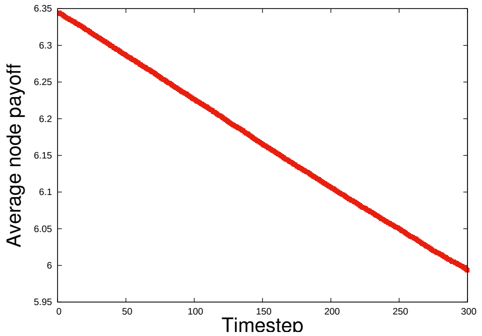

(c)

Figure 2: (a) The evolution of R-squared scale-free correlation of a network over time, when it is optimised to minimise the average KL divergence between Nash-QRE equilibria, using simulated annealing. (b) The evolution of $-\rho$, the average KL divergence between Nash-QRE equilibria, of the network over time. (c) The evolution of the average pay-off over time. Network size is $N = 20000$, $M = 40000$. The Prisoners Dilemma game was used in simulations. Note that the increase in scale-free correlation is still visible in this larger system.

For example, supplementing table 1 in the paper, we are able to report that the game parameter β makes no qualitative difference as to which topology minimises the average KL divergence between Nash and Quantal Response equilibrium. However, as Fig. 3 shows, the higher the game parameter β, the higher the difference between topologies. Also, the topology makes the greatest difference when convex functions (e.g. $f(x) = x^2$) are used in topologically modelling rationality.

This result is typical of all other experiments reported in the paper: in all cases, we found that the results do not qualitatively depend on the value of the game parameter β. However, the higher the β, the more pronounced the results. In the results presented in the paper typically $\beta = 1.67$ is used unless otherwise stated, since this value corresponds to the classic modelling of Prisoners Dilemma where the highest pay-off is 5.0 and the next highest is 3.0.

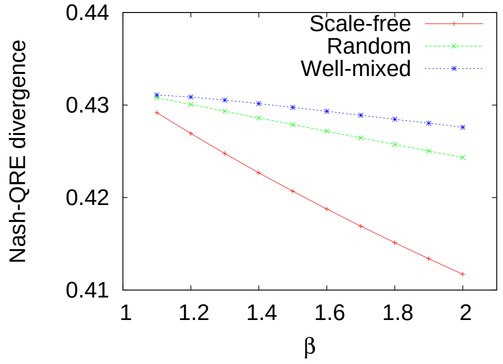

(a)

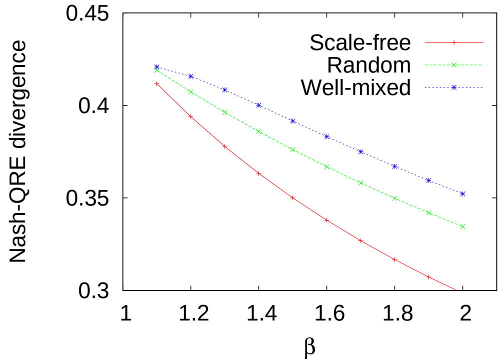

(b)

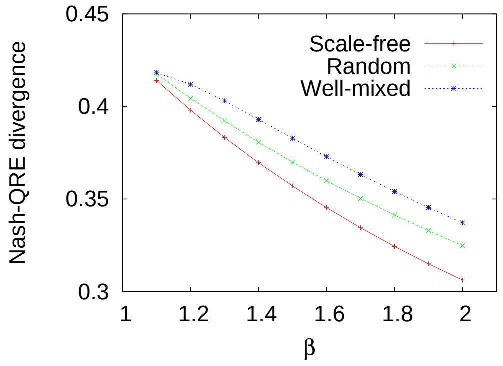

(c)

Figure 3: The average Nash-QRE divergence with varying β under (a) Convex (b) Linear and (c) Concave topological rationality functions for different topologies. The scale-free, random, and well-mixed networks are compared, and scale-free networks always show the smallest average divergence, indicating that when the systems converge towards Nash equilibrium scale-free networks emerge, regardless of the game utility values. The Prisoners Dilemma game was used in simulations. Compare with Table 1 in the paper which shows the results for two particular game parameter values.

Measuring the 'average' Kullback–Leibler divergence: an example

We use the 'average' Kullback-Leibler divergence between Nash and quantal response equilibria to quantify how 'far' the system is, on average, from that predicted by Nash, and by extension how irrational the system is. This 'average' is in fact computed in two levels, as described in methods. Let us consider a game scenario, which involves a particular pair of players in a network (there will be M such pairs, where M is the number of links). In order to use a 'symmetric' metric, we compute the 'average' Kullback-Leibler divergence between Nash and Quantal Response equilibria in each game scenario. This is computed as follows:

$$Div(N||Q) = \frac{\left(\sum_i N(i) ln \frac{N(i)}{M(i)} + \sum_i Q(i) ln \frac{Q(i)}{M(i)}\right)}{2} \tag{1}$$

where N is the probability distribution corresponding to Nash equilibrium, Q is the probability distribution corresponding to the quantal response equilibrium, and M is the distribution computed by averaging the two probability distributions N and Q. This divergence is sometimes also called the Jensen-Shannon divergence.

For example, for the Prisoner's Dilemma game, it is well known that the Nash equilibrium would be the state where both players defect. Thus the probability distribution at Nash equilibrium would be $N = \{p_1^c, p_1^d, p_2^c, p_2^d\} = \{0, 1, 0, 1\}$. The cooperation probability is zero and the defection probability is one for both players. Suppose the Quantal response equilibrium for a particular rationality parameter for a pair of players connected by a link is computed as $N = \{p_1^c, p_1^d, p_2^c, p_2^d\} = \{0.3, 0.7, 0.4, 0.6\}$. Then, the average probability distribution be-

tween N and Q would be $M = \{0.15, 0.85, 0.2, 0.8\}$. Thus, the resulting Jensen-Shannon divergence metric value would be computed as:

$$KL(N||M) = 0 + 1.ln\frac{1}{0.85} + 0 + 1.ln\frac{1}{0.8} = 0.3857 \tag{2}$$

$$KL(Q||M) = 0.3.ln\frac{0.3}{0.15} + 0.7.ln\frac{0.7}{0.85} + 0.4.ln\frac{0.4}{0.2} + 0.6.ln\frac{0.6}{0.8} = 0.1767 \tag{3}$$

Thus:

$$Div(N||Q) = (0.3857 + 0.1767)/2.0 = 0.2812 \tag{4}$$

for this particular pair of players. All such pairs (M of them since this is the number of links) are then further averaged to compute a network-level average divergence value ρ, such that

$$\rho = -\frac{1}{M}\sum_{k=1}^{M} Div(N_k||Q_k) \tag{5}$$

We insert the minus sign to indicate that the smaller this divergence, the higher the system rationality. Note that we have chosen to use the natural logarithm in all our computations including the above example.

Relationship between rationality and social interaction: an extended explanation

We have argued in the paper that there is an implicit relationship between the amount of social interaction of a particular player and their bounded rationality. This argument is critical in topologically quantifying the bounded rationality of players. Indeed, a number of studies have articulated this view. For instance, the social cognitive theory [1] identifies five core concepts in modelling the social perspectives of cognition; observational learning, outcome expectations, self-efficacy, goal setting and self regulation. The first aspect, which is observational learning, suggests that knowledge acquisition is directly correlated to the observation of models. All these aspects can be related to strategic decision making environments, where the players may learn from each other. Thus, a player with a relatively higher amount of social interactions may have higher cognitive capacity compared to a player with a relatively lower amount of social interactions. The social learning theory [2] expands on this concept by identifying four key factors in learning new behaviour; drives, cues, responses and rewards. It could be argued that the abundance of these factors increases with the amount of social interactions of each player, in turn increasing their cognitive capacity.

The social brain hypothesis [3,4] provides another interesting avenue to theorise how the social influence, or the social group size of an individual, may reflect upon the cognitive capacity of that individual. Extending on this hypothesis, studies have been conducted which establish that there is a correlation between the human brain cortex size and the social cognitive capacity of humans [5]. Conventionally, it was assumed that the relatively large brain size of humans was due to the evolutionary advantage that it provided in collecting and processing

information. However, social brain hypothesis suggests that the larger brain size of primates and humans in general is correlated to the complex and large social groups that they form. Indeed, it could also be argued that the cognitive capacity of a person (player) is an inherent property of a person, and the amount of interactions he engages in is rather a reflection of that cognitive capacity. Either way, based on the above mentioned studies, it is reasonable to argue that the cognitive capacity, i.e rationality, of a player is positively correlated to the amount of social interactions they undertake.

1. Bandura, A. Social cognitive theory: An agentic perspective. *Annual review of psychology* **52**, 1–26 (2001).
2. Bandura, A. & McClelland, D. C. Social learning theory (1977).
3. Dunbar, R. I. The social brain hypothesis. *brain* **9**, 178–190 (1998).
4. Dunbar, R. I. The social brain hypothesis and its implications for social evolution. *Annals of human biology* **36**, 562–572 (2009).
5. Powell, J. L., Lewis, P. A., Dunbar, R. I., García-Fiñana, M. & Roberts, N. Orbital prefrontal cortex volume correlates with social cognitive competence. *Neuropsychologia* **48**, 3554–3562 (2010).
